# Supplementary material for: Variant interpretation using population databases: Lessons from gnomAD
Source: Hum Mutat. 2021 Dec 16;43(8):1012–30. doi: 10.1002/humu.24309 (PMC9160216; doi:10.1002/humu.24309)
Supplement: Supplementary file 1 — Supplementary information. [file HUMU-43-1012-s001.docx]

# **SUPPLEMENTARY DATA: Variant interpretation using population databases: lessons from gnomAD**

Sanna Gudmundsson^1,2,3^, Moriel Singer-Berk^1,3^, Nicholas A. Watts^1,3^, William Phu^1,2,3^, Julia K. Goodrich^1,3^, Matthew Solomonson^1,3^, Genome Aggregation Database Consortium, Heidi L. Rehm^1,3,4^, Daniel G. MacArthur^1,5,6^, Anne O’Donnell-Luria^1,2,3^

1. Program in Medical and Population Genetics, Broad Institute of MIT and Harvard, Cambridge, MA, USA

2. Division of Genetics and Genomics, Boston Children's Hospital, Harvard Medical School, Boston, MA, USA

3. Analytic and Translational Genetics Unit, Massachusetts General Hospital, Boston, MA, USA

4. Center for Genomic Medicine, Massachusetts General Hospital, Boston, MA, USA

5. Centre for Population Genomics, Garvan Institute of Medical Research, and University of New South Wales Sydney, Sydney, Australia

6. Centre for Population Genomics, Murdoch Children’s Research Institute, Melbourne, Australia

## **Supplementary methods**

**Analysis of very rare coding variants in gnomAD populations**

A sample of 100 individuals was randomly selected from each of the following populations in the gnomAD v2.1.1 exome dataset: African/African American, Latino/Admixed American, East Asian, Non-Finnish European, and South Asian. Variants were then filtered to only very rare variants using a popmax allele frequency of < 0.1% (popmax refers to the maximum frequency across continental populations in gnomAD, not including Ashkenazi Jewish, Finnish, or any other remaining samples) across the entire gnomAD dataset (v2 exomes, v2 genomes, and v3 genomes). Variants were also excluded if they had not passed gnomAD quality control or had been flagged as part of a problematic region (low complexity, decoy, and segmental duplication). Unique variants for the 100 individuals were obtained by filtering to variants with AC of 1 in gnomAD v2 exomes and no observations in v2 genomes or in the v3-non v2 subset.

The variant annotations previously applied by the Variant Effect Predictor (VEP; version 85) were used to assign each variant to one of three categories: pLoF, missense/inframe indel, or synonymous [(McLaren et al., 2016)](https://paperpile.com/c/tjMujv/dPHL). The VEP annotations were filtered to the most severe consequence for the canonical transcript. A variant was annotated as pLoF if the most severe consequence was one of: "splice_acceptor", "splice_donor_variant", "stop_gained", "frameshift_variant" and it was found to be high-confidence (indicated by LOFTEE) [(Karczewski et al., 2020)](https://paperpile.com/c/tjMujv/xpKA). The missense/indel annotation was applied if the most severe consequence was one of: "missense_variant", "inframe_insertion", "inframe_deletion". A synonymous annotation was used for a variant if the most severe consequence was "synonymous_variant". Analysis was performed using Hail (<https://hail.is/>). Variants were exported to a tsv for data visualization in R. The code used to select individuals and filter variants can be found on GitHub: <https://github.com/broadinstitute/gnomad_review_hum_mut>.

## **Supplementary figures and tables**

**Supplementary Table S1:** Mean count of very rare coding variants per population (allele frequency < 0.1%). Total unique refers to variants found in only that individual across the entire gnomAD dataset (v2 exome, v2 genome, v3).

| **Population** | **pLoF** | **Missense/ indel** | **Synonymous** | **Total** | **Total unique** |
| --- | --- | --- | --- | --- | --- |
| **African/African American** | 7 (±3) | 137 (±18) | 77 (±12) | 220 (±27) | 21 (±7) |
| **Latino/Admixed American** | 6 (±3) | 111 (±20) | 57 (±12) | 175 (±29) | 19 (±11) |
| **East Asian** | 7 (±3) | 127 (±21) | 66 (±13) | 201 (±32) | 35 (±11) |
| **European (non-finnish)** | 7 (±2) | 108 (±18) | 55 (±13) | 170 (±30) | 23 (±11) |
| **South Asian** | 7 (±3) | 147 (±22) | 80 (±14) | 235 (±33) | 38 (±14) |
| **Global** | **7 (±3)** | **126 (±25)** | **67 (±16)** | **200 (±39)** | **27 (±13)** |

(±) standard deviation

**Supplementary Table S2:** Comparison of mean count of unique coding variants per population utilizing only v2 exome dataset or the entire gnomAD dataset. The general decrease in the number of unique variants from mean 36 (± 16) to 27 (± 13) (p < 2.2e-16) across populations demonstrates the importance of increased sample size and more diverse representation of populations.

| **Population** | **v2 exome only** | **Entire gnomAD** | **P-value** |
| --- | --- | --- | --- |
| **African/African American** | 42 (±11) | 21 (±7) | 2.20E-16 |
| **Latino/Admixed American** | 26 (±18) | 19 (±11) | 3.49E-03 |
| **East Asian** | 42 (±13) | 35 (±11) | 7.85E-05 |
| **European (non-Finnish)** | 29 (±12) | 23 (±11) | 1.52E-04 |
| **South Asian** | 41 (±15) | 38 (±14) | 9.46E-02 |
| **Global** | **36 (±16)** | **27 (±13)** | **2.20E-16** |

P-value calculated using Student t-test.

**
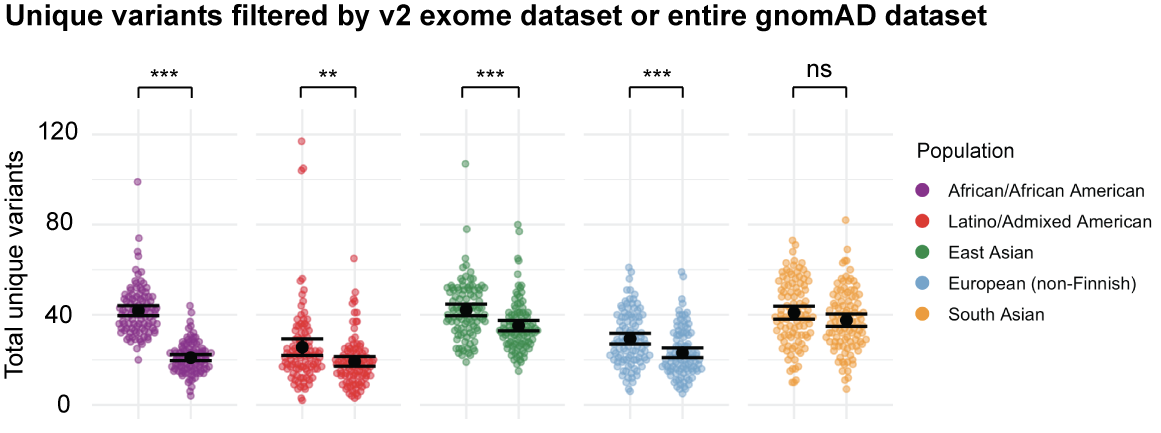
**

**Supplementary Figure S1:** Total unique variants in two sets of 100 randomly selected individuals filtered using v2 exomes (n = 125,748; left) versus the entire gnomAD dataset (n = 197,912; right). The increased sample size results in a decrease in the number of unique variants per individual in all populations. The increase from ~8,000 African/African American individuals in v2 exomes to ~30,000 individuals across the entire database results in a significant decrease in unique variants from mean 42 (± 11) to 21 (± 7) variants per individual in this population (purple). In comparison the minor increase in South Asian samples from ~15,000 to ~16,500 results in an non-significant decrease from mean 41 (± 15) to 38 (± 14) variants per individual (orange). Of note, the mean is influenced by the fact that these individuals are in gnomAD and likely from a community that has some representation in the database. An individual from a subpopulation or community that is not as well represented in gnomAD will likely have a higher number of unique very rare variants than seen in this analysis. Black bar represents the 95% confidence interval. ** p < 0.01, *** p < 0.001, ns: not significant with Student t-test. (±) Standard deviation.


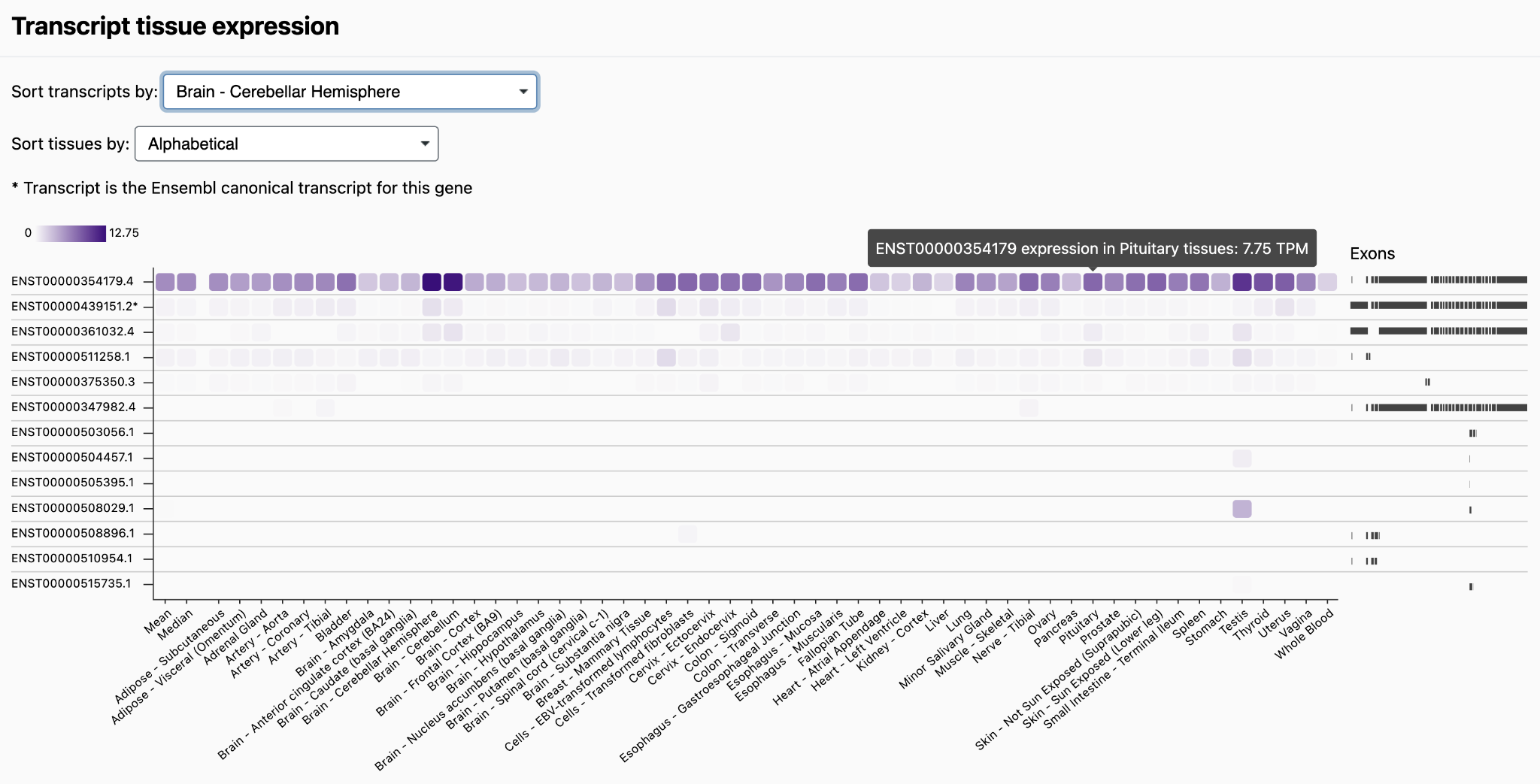


**Supplementary Figure S2:** Transcript tissue expression of *NSD1*. Navigate to the pop-up window by selecting “Show transcripts” (Figure 3:6) or “Show tissues” (Figure 3:11) on the gene page, and then “show transcript tissue expression” (Figure 3:8). Transcripts (y-axis) can be sorted by their expression in a specific tissue. In this example, sorting by Brain - Cerebellar Hemisphere lists ENST00000354179.4 first as the highest expressed transcript in that tissue. Tissues (x-axis) can be sorted alphabetically (this example) or by highest mean expression. A darker purple square indicates higher expression of a specific transcript in a specific tissue measured by transcripts per million (TPM). The exact value is available as a hover over (in this example highlighting the expression of ENST00000354179.4 in Pituitary tissue as 7.75 TPM). The highest expression is found in “Brain - Cerebellar Hemisphere” and “Brain - Cerebellum” (darkest purple). *The canonical transcript.


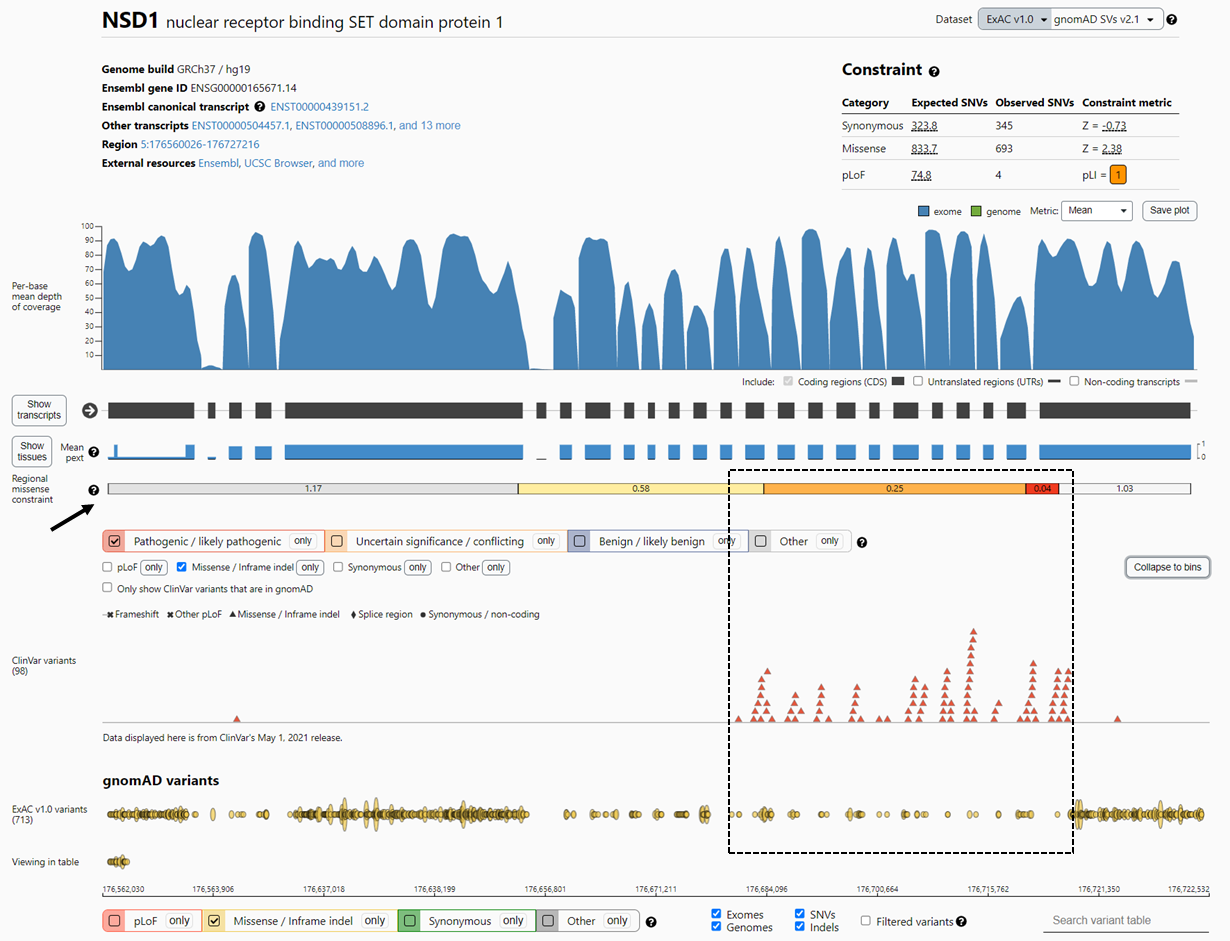


**Supplementary Figure S3:** ExAC dataset gene page for *NSD1*. The track is available under the pext track (black arrow) for genes with regional missense constraint. The numbers on the track refer to the proportion of observed versus expected missense variation seen in the region in gnomAD. In this example, missense constrained regions (red and orange) show clustering of pathogenic/likely pathogenic missense variants in ClinVar, while there is a depletion of missense variation in gnomAD for the same region (box).


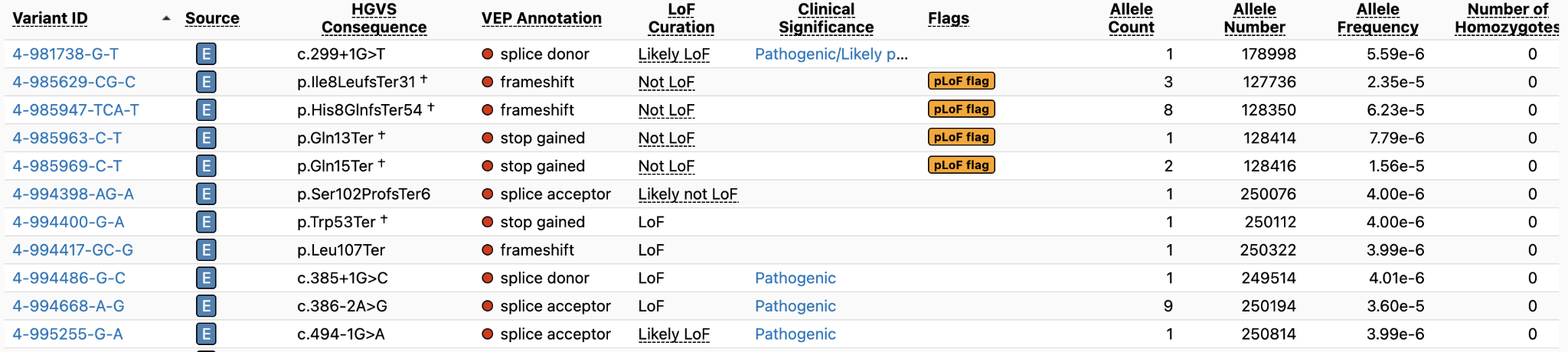


**Supplementary Figure S4:** Variant table from *IDUA* showing the manual curation verdict (under LoF Curation), the LoF transcript effect estimator (LOFTEE) low-confidence (LC) or pLoF flag (under Flags), with further detail supplied on the variant page. Generally, manual curation was not necessary or performed if a variant was determined to be LC by LOFTEE. Manual curation verdicts are LoF, Likely LoF, Uncertain LoF, Likely not LoF, and Not LoF.

**
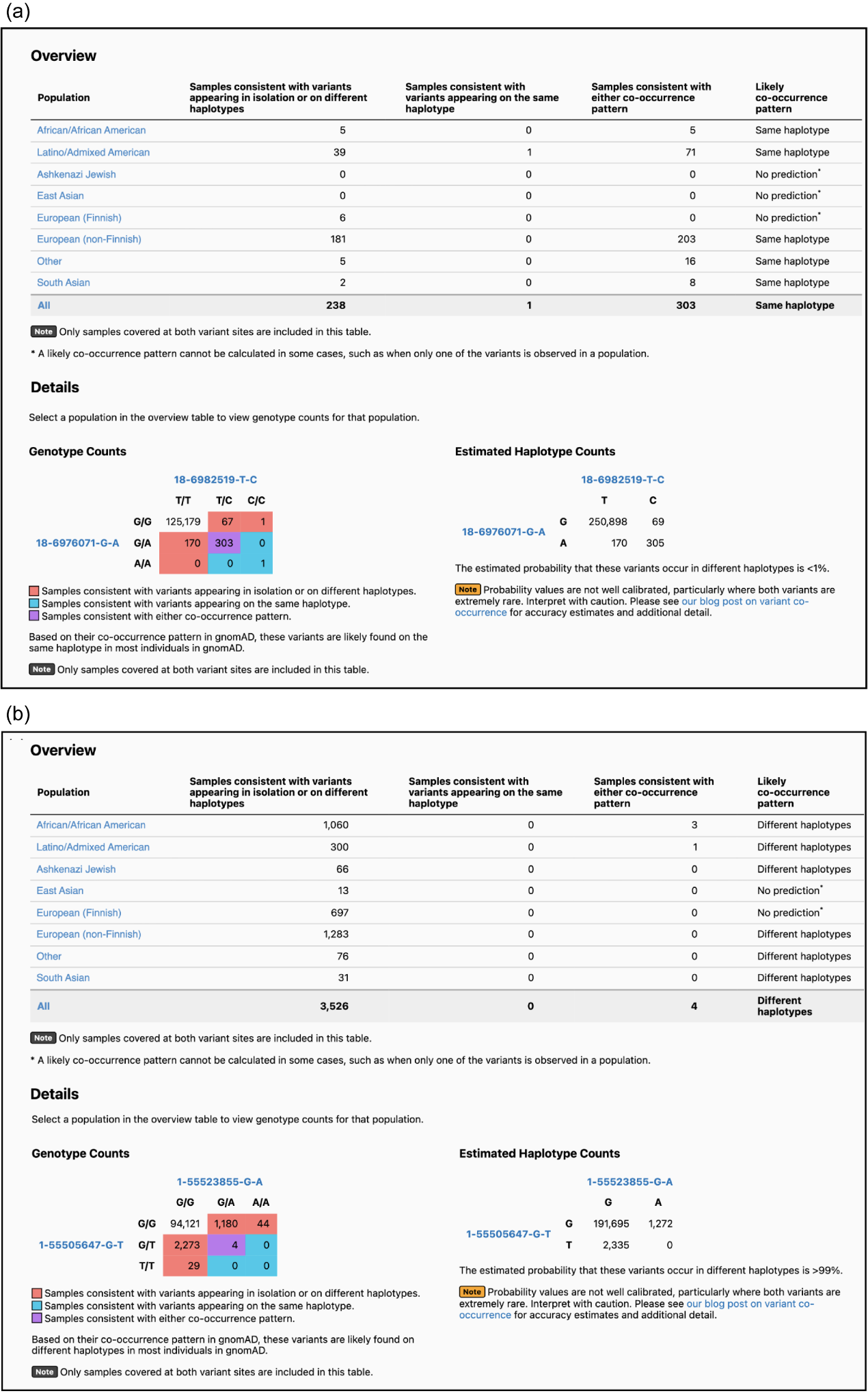
**

**Supplementary Figure S5:** The variant co-occurrence feature shows the probability that two variants of interest are found on different haplotypes using a statistical phasing approach. (A) In the first example chr18:6976071G>A (NM_005559.4:c.6354C>T) and chr18:6982519T>C (NM_005559.4:c.5867A>G) in the *LAMA1* gene are investigated. The variants are found in isolation in 68 and 170 individuals (red) and co-occur in 304 individuals (purple and blue). Given the high proportion of individuals where both variants are present, they are predicted to be on the same haplotype in the informative populations. East Asian and European (Finnish) are uninformative as there are no individuals harboring both variants in these populations. The variants must be on the same haplotype in the one individual (blue) who is homozygous for both variants. (B) In the second example chr1:55505647G>T (NM_174936.4:c.137G>T) and chr1:55523855G>A (NM_174936.4:c.1327G>A) in the *PCSK9* gene are investigated. They are reported to likely be on different haplotypes in all informative populations as they co-occur in only four individuals in gnomAD (purple). Thus, the presence of these four individuals in gnomAD suggest that compound heterozygosity for these two variants is not associated with a severe, early-onset, highly penetrant disorder.


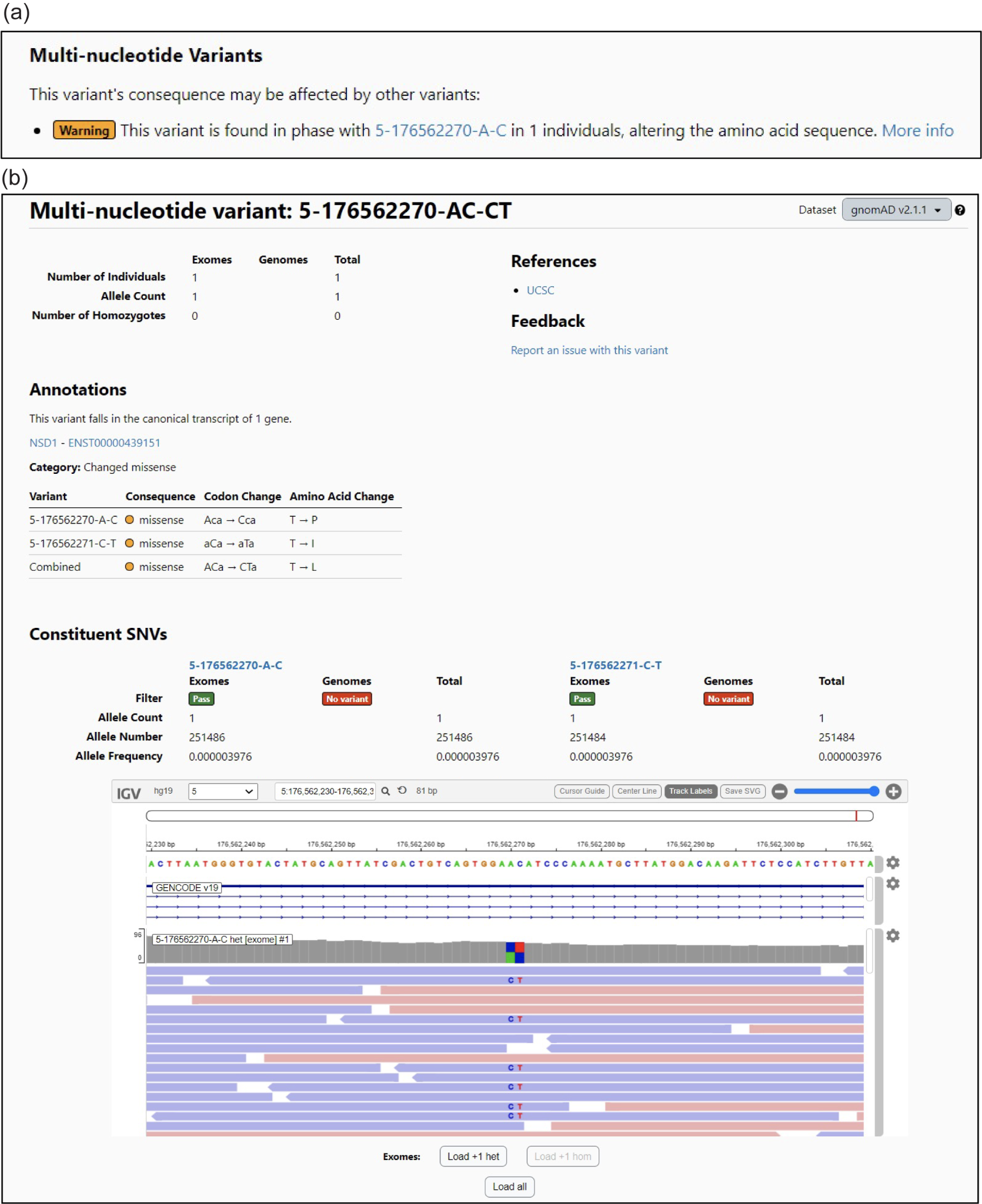


**Supplementary Figure S6:** Multi-nucleotide variant (MNV) in *MBD5*. (a) From the variant page for either of the individual variants, the warning indicates that the variant is part of an MNV. (b) The header for the MNV page displays frequency information for the MNV. The predicted variant type of each separate variant, as well as for the combined variant type is noted in the table (in this case a stop-gained and missense variant within this codon in combination produces a missense variant). The variant links navigate directly to the page for each variant.


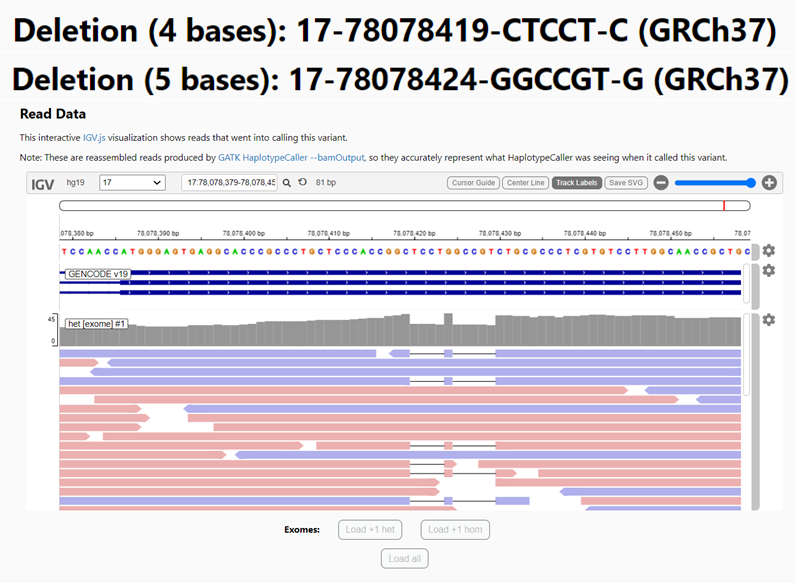


**Supplementary Figure S7:** Frame-restoring indel in the *GAA* gene. While each variant interpreted independently would result in a frameshift, in combination these result in a single 9 base-pair (3 amino acid) in-frame deletion. The effect of the variant is not LoF and should be interpreted as an in-frame indel. Indels (either as SNV-indel variants or indel-indel variants) are not currently annotated in the gnomAD browser as part of MNVs.


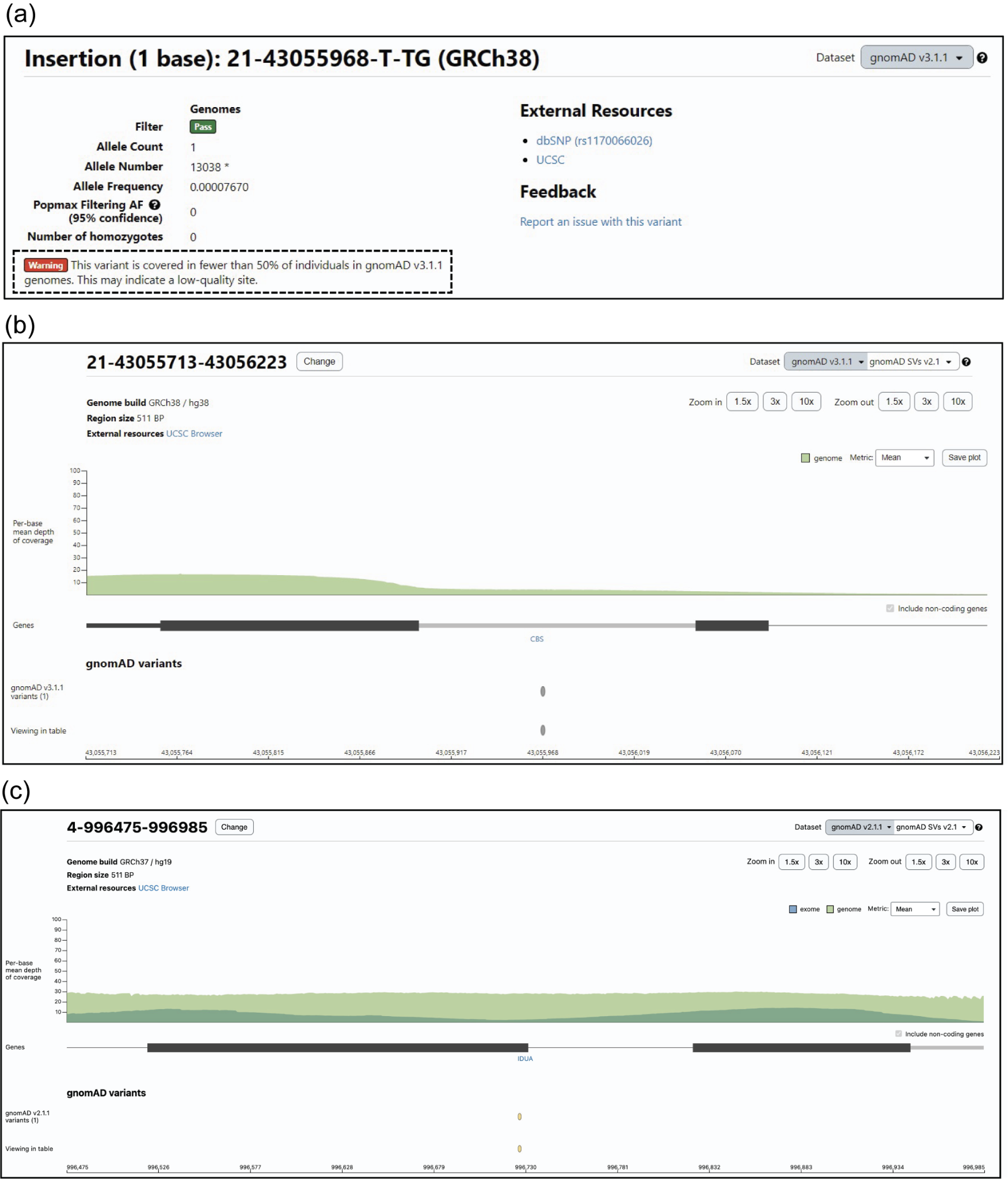


**Supplementary Figure S8:** Low coverage warning and caveats. (a) Top panel displays a warning (dashed box) that the variant in the *CBS* gene is covered in fewer than 50% of individuals. (b) Regional view of the *CBS* gene (searching for an interval in the search box) displaying low coverage in genomes (gnomAD v3) for the region of the highlighted variant (gray oval). (c) Regional view of *IDUA* gene displaying low coverage in exomes (blue), but not genome (green) in gnomAD v2 for the region of the highlighted variant (yellow oval). Allele frequencies in the region are likely reliable in the *IUDA* gene from the genome (but not exome) data (c) but not reliable in *CBS* gene from either genome or exome data (b).


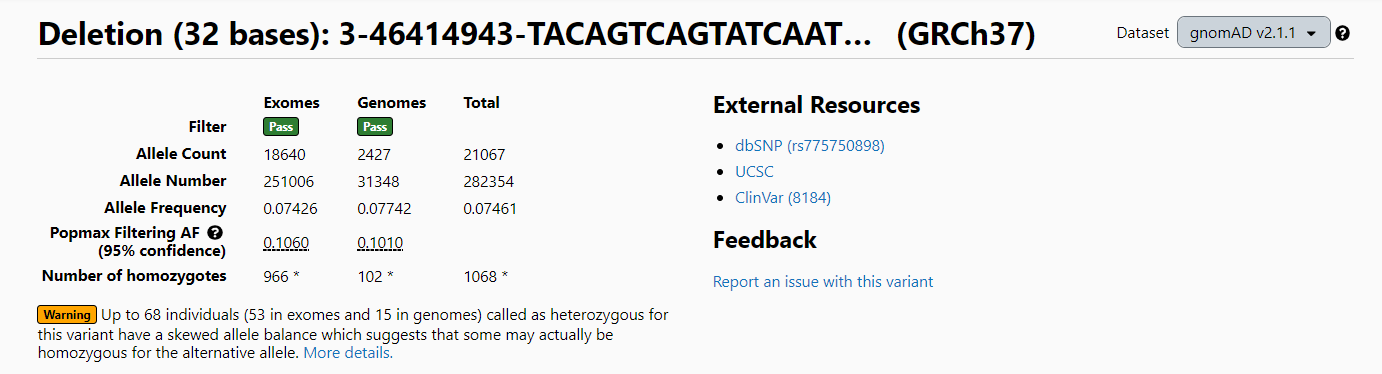


**Supplementary Figure S9:** Flag for variant with a skewed allele balance for homozygous variants. Heterozygous variants with inflated alt-reads are likely homozygous variants called heterozygotes due to contamination that affects the variant calling likelihood models. This depletion of homozygous calls can incorrectly deflate the allele frequencies [(Karczewski et al., 2019)](https://paperpile.com/c/tjMujv/QRuo).

## **References**

[McLaren, W., Gil, L., Hunt, S. E., Riat, H. S., Ritchie, G. R. S., Thormann, A., … Cunningham, F. (2016). The Ensembl Variant Effect Predictor. *Genome Biology*, *17*(1), 122.](http://paperpile.com/b/tjMujv/dPHL)

[Karczewski, K. J., Francioli, L. C., Tiao, G., Cummings, B. B., Alföldi, J., Wang, Q., … MacArthur, D. G. (2020). The mutational constraint spectrum quantified from variation in 141,456 humans. *Nature*, *581*(7809), 434–443.](http://paperpile.com/b/tjMujv/xpKA)

[Karczewski, K. J., Gauthier, L. D., & Daly, M. J. (2019). Technical artifact drives apparent deviation from Hardy-Weinberg equilibrium at CCR5-∆32 and other variants in gnomAD (p. 784157). https://doi.org/](http://paperpile.com/b/tjMujv/QRuo)[10.1101/784157](http://dx.doi.org/10.1101/784157)

## **Genome Aggregation Database Consortium funding statements**

Matthew J. Bown: British Heart Foundation awards CS/14/2/30841 and RG/18/10/33842

Josée Dupuis: National Heart Lung and Blood Institute's Framingham Heart Study Contract (HHSNI); National Institute for Diabetes and Digestive and Kidney Diseases (NIDDK) R DK

Martti Färkkilä: State funding for university level health research

Laura D. Gauthier: Intel, Illumina

Stephen J. Glatt: U.S. NIMH Grant R MH

Leif Groop: The Academy of Finland and University of Helsinki: Center of Excellence for Complex Disease Genetics (grant number 312063 and 336822), Sigrid Jusélius Foundation; IMI 2 (grant No 115974 and 15881 )

Mikko Hiltunen: Academy of Finland (grant 338182) Sigrid Jusélius Foundation the Strategic Neuroscience Funding of the University of Eastern Finland

Chaim Jalas: Bonei Olam

Jaakko Kaprio: Academy of Finland (grants 312073 and 336823)

Jacob McCauley: National Institute of Diabetes and Digestive and Kidney Disease Grant R01DK104844

Yukinori Okada: JSPS KAKENHI (19H01021, 20K21834), AMED (JP21km0405211, JP21ek0109413, JP21gm4010006, JP21km0405217, JP21ek0410075), JST Moonshot R&D (JPMJMS2021)

Michael J. Owen: Medical Research Council UK: Centre Grant No. MR/L010305/1, Program Grant No. G0800509

Aarno Palotie: the Academy of Finland Center of Excellence for Complex Disease Genetics (grant numbers 312074 and 336824) and Sigrid Jusélius Foundation

John D. Rioux: National Institute of Diabetes and Digestive and Kidney Diseases (NIDDK; DK062432), from the Canadian Institutes of Health (CIHR GPG 102170), from Genome Canada/Génome Québec (GPH-129341), and a Canada Research Chair (#230625)

Samuli Ripatti: the Academy of Finland Center of Excellence for Complex Disease Genetics (grant number ) Sigrid Jusélius Foundation

Jerome I. Rotter: Trans-Omics in Precision Medicine (TOPMed) program was supported by the National Heart, Lung and Blood Institute (NHLBI). WGS for “NHLBI TOPMed: Multi-Ethnic Study of Atherosclerosis (MESA)” (phs001416.v1.p1) was performed at the Broad Institute of MIT and Harvard (3U54HG003067-13S1). Core support including centralized genomic read mapping and genotype calling, along with variant quality metrics and filtering were provided by the TOPMed Informatics Research Center (3R01HL-117626-02S1; contract HHSN268201800002I). Core support including phenotype harmonization, data management, sample-identity QC, and general program coordination were provided by the TOPMed Data Coordinating Center (R01HL-120393; U01HL-120393; contract HHSN268201800001I). We gratefully acknowledge the studies and participants who provided biological samples and data for MESA and TOPMed. JSK was supported by the Pulmonary Fibrosis Foundation Scholars Award and grant K23-HL-150301 from the NHLBI. MRA was supported by grant K23-HL-150280, AJP was supported by grant K23-HL-140199, and AM was supported by R01-HL131565 from the NHLBI. EJB was supported by grant K23-AR-075112 from the National Institute of Arthritis and Musculoskeletal and Skin Diseases.The MESA project is conducted and supported by the National Heart, Lung, and Blood Institute (NHLBI) in collaboration with MESA investigators. Support for MESA is provided by contracts 75N92020D00001, HHSN268201500003I, N01-HC-95159, 75N92020D00005, N01-HC-95160, 75N92020D00002, N01-HC-95161, 75N92020D00003, N01-HC-95162, 75N92020D00006, N01-HC-95163, 75N92020D00004, N01-HC-95164, 75N92020D00007, N01-HC-95165, N01-HC-95166, N01-HC-95167, N01-HC-95168, N01-HC-95169, UL1-TR-000040, UL1-TR-001079, and UL1-TR-001420. Also supported in part by the National Center for Advancing Translational Sciences, CTSI grant UL1TR001881, and the National Institute of Diabetes and Digestive and Kidney Disease Diabetes Research Center (DRC) grant DK063491 to the Southern California Diabetes Endocrinology Research Center

Edwin K. Silverman: NIH Grants U01 HL089856 and U01 HL089897

J. Gustav Smith: The Swedish Heart-Lung Foundation (2019-0526), the Swedish Research Council (2017-02554), the European Research Council (ERC-STG-2015-679242), Skåne University Hospital, governmental funding of clinical research within the Swedish National Health Service, a generous donation from the Knut and Alice Wallenberg foundation to the Wallenberg Center for Molecular Medicine in Lund, and funding from the Swedish Research Council (Linnaeus grant Dnr 349-2006-237, Strategic Research Area Exodiab Dnr 2009-1039) and Swedish Foundation for Strategic Research (Dnr IRC15-0067) to the Lund University Diabetes Center

Kent D. Taylor: Trans-Omics in Precision Medicine (TOPMed) program was supported by the National Heart, Lung and Blood Institute (NHLBI). WGS for “NHLBI TOPMed: Multi-Ethnic Study of Atherosclerosis (MESA)” (phs001416.v1.p1) was performed at the Broad Institute of MIT and Harvard (3U54HG003067-13S1). Core support including centralized genomic read mapping and genotype calling, along with variant quality metrics and filtering were provided by the TOPMed Informatics Research Center (3R01HL-117626-02S1; contract HHSN268201800002I). Core support including phenotype harmonization, data management, sample-identity QC, and general program coordination were provided by the TOPMed Data Coordinating Center (R01HL-120393; U01HL-120393; contract HHSN268201800001I). We gratefully acknowledge the studies and participants who provided biological samples and data for MESA and TOPMed. JSK was supported by the Pulmonary Fibrosis Foundation Scholars Award and grant K23-HL-150301 from the NHLBI. MRA was supported by grant K23-HL-150280, AJP was supported by grant K23-HL-140199, and AM was supported by R01-HL131565 from the NHLBI. EJB was supported by grant K23-AR-075112 from the National Institute of Arthritis and Musculoskeletal and Skin Diseases.The MESA project is conducted and supported by the National Heart, Lung, and Blood Institute (NHLBI) in collaboration with MESA investigators. Support for MESA is provided by contracts 75N92020D00001, HHSN268201500003I, N01-HC-95159, 75N92020D00005, N01-HC-95160, 75N92020D00002, N01-HC-95161, 75N92020D00003, N01-HC-95162, 75N92020D00006, N01-HC-95163, 75N92020D00004, N01-HC-95164, 75N92020D00007, N01-HC-95165, N01-HC-95166, N01-HC-95167, N01-HC-95168, N01-HC-95169, UL1-TR-000040, UL1-TR-001079, and UL1-TR-001420. Also supported in part by the National Center for Advancing Translational Sciences, CTSI grant UL1TR001881, and the National Institute of Diabetes and Digestive and Kidney Disease Diabetes Research Center (DRC) grant DK063491 to the Southern California Diabetes Endocrinology Research Center

Tiinamaija Tuomi: The Academy of Finland and University of Helsinki: Center of Excellence for Complex Disease Genetics (grant number 312072 and 336826 ), Folkhalsan Research Foundation, Helsinki University Hospital, Ollqvist Foundation, Liv och Halsa foundation; NovoNordisk Foundation

Teresa Tusie-Luna: CONACyT Project 312688

James S. Ware: Wellcome Trust [107469/Z/15/Z], Medical Research Council (UK), NIHR Imperial College Biomedical Research Centre

Rinse K. Weersma: The Lifelines Biobank initiative has been made possible by subsidy from the Dutch Ministry of Health Welfare and Sport the Dutch Ministry of Economic Affairs the University Medical Centre Groningen (UMCG the Netherlands ) the University of Groningen and the Northern Provinces of the Netherlands
